# The phenomena of balanced effect between α-globin gene and of β-globin gene

**DOI:** 10.1186/s12881-018-0659-9

**Published:** 2018-08-17

**Authors:** Liangying Zhong, Xin Gan, Lingling Xu, Chujia Liang, Yingjun Xie, Wenbin Lin, Peisong Chen, Min Liu

**Affiliations:** 1 Department of Laboratory Medicine, The First Affiliated Hospital, Sun Yat-sen University, Guangzhou, 510080 Guangdong China; 2 Department of Respiratory Medicine, The First Affiliated Hospital of Nanchang University, Nanchang, 330006 China; 3 Department of Pediatrics, The First Affiliated Hospital, Sun Yat-sen University, Guangzhou, 510080 China; 4 Key Laboratory for Major Obstetric Diseases of Guangdong Province, Key Laboratory of Reproduction and Genetics of Guangdong Higher Education Institutes, The Third Affiliated Hospital of Guangzhou Medical University, Guangzhou, 510150 China

**Keywords:** Thalassemia intermedia-Deletional Hb H-β^0^-thalassemia (β^0^-thal)-β^+^-thalassemia (β^+^-thal)

## Abstract

**Background:**

Thalassemias (TM) are the most common autosomal recessive disorders in Southeast Asian countries. Both α- and β-thalassemia lead to a decrease or absence of globin chains. The most serious of the thalassemia syndromes is thalassemia major which is characterized by a transfusion dependent anemia and subsequent iron overload caused by repeated blood transfusions. It is preventive by genotyping the parents. A better understanding of the laboratory data will help provide an accurate diagnosis of thalassemia major, and prevention and controlling programs in routine laboratories.

**Case presentation:**

The patient was a one-year-old boy born to non-consanguineous parents. He was referred to our outpatient clinic for hemolytic anemia after a cold. Hematological investigations revealed severe anemia (Hb57 g/dL). The red cells displayed microcytosis, hypochromia and misshapen erythrocytes (MCV48.8 fL, MCH15.7 pg). Capillary electrophoresis (CE) electropherogram revealed normal level of HbA2 (3.2%) and elevated HbF (35.1%). The patient was diagnosed with β-TM, based on severe microcytosis, hypochromia, normal Hb A2 and high Hb F level but no Hb H inclusion at the first visit. Later our molecular analysis revealed compound heterozygosity for codons 41–42 (-TTCT) (HBB: c.126_129delCTTT, β^0^) and IVS-II-654 (C > T) (HBB: c.316-197C > T, β^+^) mutation and deletional Hb H (−-^SEA^/−α^3.7^). Thus, a combination of Hb H disease and a compound heterozygosity of β^+^/β^0^-thalassemia (β^+^/β^0^-thal) was finally diagnosed.

**Conclusions:**

Genotype-phenotype analysis shows that heterozygous mutations in the β-globin gene could affect not only hematological parameters, but also elevate HbA2 levels. These effects could be ameliorated by the coinheritance of Hb H disease, which may be explained by the phenomena of the α-globin gene and of the β-globin gene balanced effect.

**Electronic supplementary material:**

The online version of this article (10.1186/s12881-018-0659-9) contains supplementary material, which is available to authorized users.

## Background

Beta-thalassemia is characterized by a reduced or absent synthesis of the β-globin chain of hemoglobin. It is an autosomal recessive disorder in Southern China with an incidence rate of 2.54% in Guangdong [1] and 6.78% in Guangxi provinces [2]. According to genotype, clinical symptoms, as well as transfusion needs, β-thalassemia includes three main forms: (1) Mild or asymptomatic thalassemia. It is also called “β-thalassemia carrier” or “heterozygous β-thalassemia”. This group of patients present with hemoglobin (Hb) levels at 9–12 g/dL, and usually shows mild anemia or asymptomatic in early age. (2) Moderate thalassemia. This group of thalassemia is mostly caused by homozygous or compound heterozygous mutations. The Hb level is maintained between 6 and 7 g/dL. No blood transfusion is required unless the patients developed infections which worsen the anemia. (3) Severe thalassemia. It is also referred to “Cooley’s Anemia”. Severe thalassemia is blood transfusion-dependent from infancy for survival, and the patients are homozygotes or compound heterozygotes for β^0^ or β^+^ genes. The Hb level is minimal and can be as less as 4–5 g/dL [3–6]. Hemoglobin H (Hb H) disease is caused by an absence or diminished synthesis of the α-globin chain of the hemoglobin molecule. Generally, Hb H disease can be classified as deletional and non-deletional. The deletional form is caused by the deletions of three α-globin genes (−−/−α) while the latter one is caused by the --^SEA^ deletion with α -globin variant.

Compound heterozygosity of two β-thalassemia genes mutations usually results in severe Cooley’s Anemia disease [4]. To the best of our knowledge, there are few reports of coinheritance of Hb H disease with compound heterozygosity of β-thalassemia except for a case that previously reported the co-existence of Hb H disease gmx(−-^SEA^/−α^4.2^) and β-thalassemia major in the Chinese population [7]. Here, we report another case of Hb H disease (−-^SEA^/−α^3.7^) combined with compound heterozygosity of IVS-II -654(C > T) and codons 41–42 (-TTCT) in the Chinese population with the genotype–phenotype correlation analyses.

## Case presentation

**Fig. 1:**
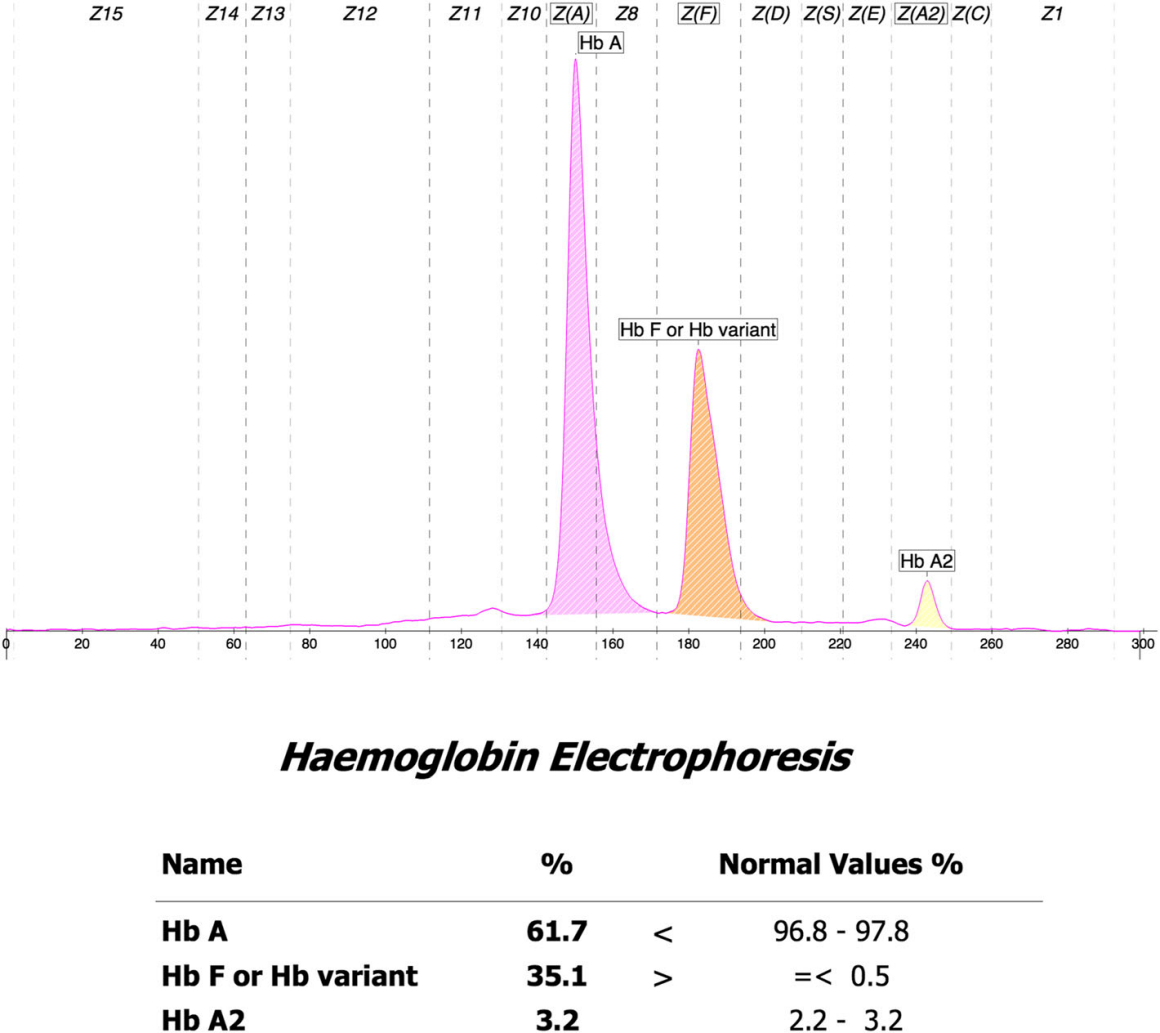
The percentages of HbA2 and Hb F were 3.2 and 35.1%, respectively by the hemoglobin electrophoresis

**Fig. 2:**
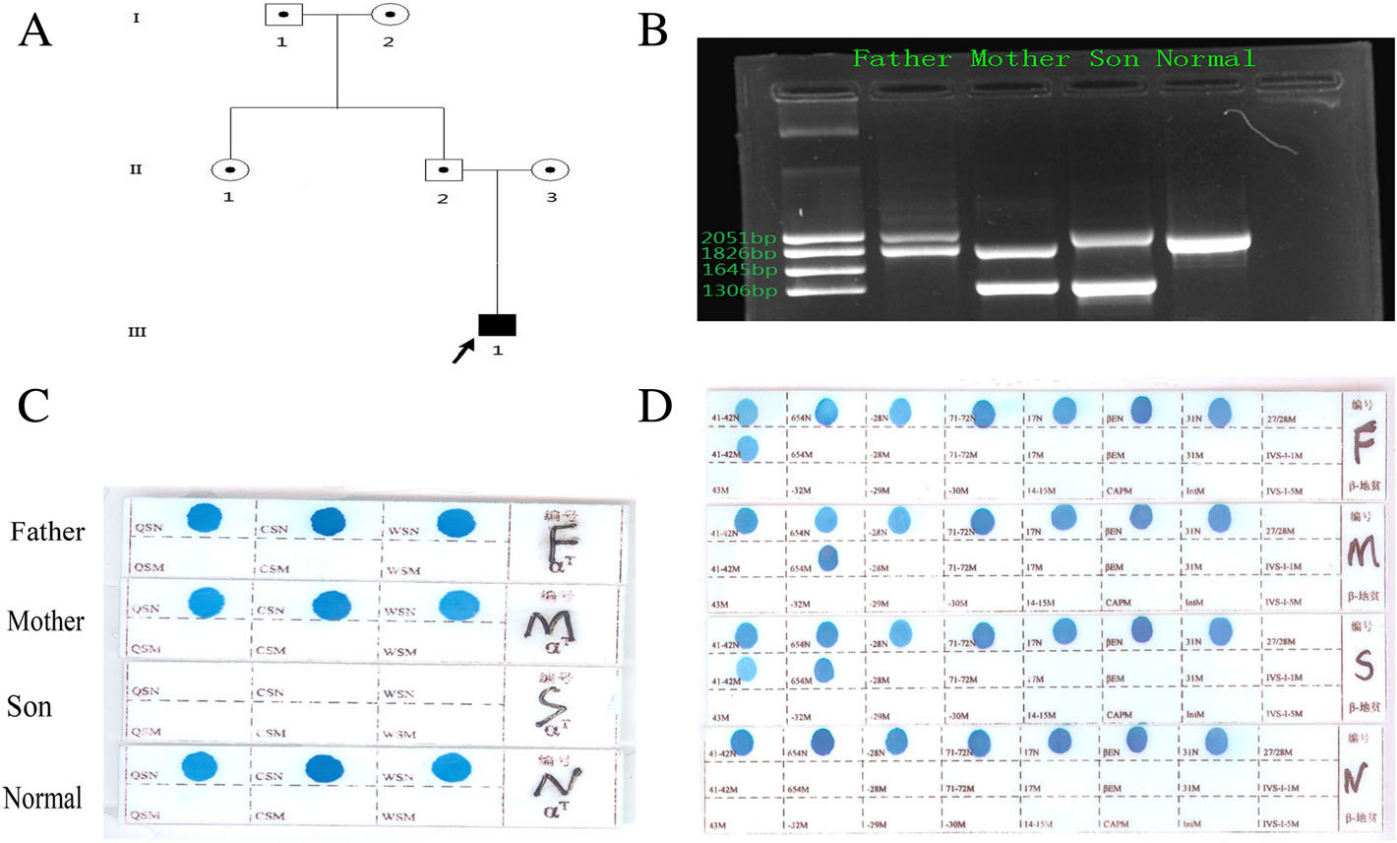
**a**. Pedigree of the family with Hb H (−α^3.7^/−-^SEA^) and β^CD41–42^ /β^IVS-II-654^. The rightward deletion (−α^3.7^) was combined with codons 41–42 (-TTCT) mutations in the β-globin gene in the proband’s father (II:2), a Southeastern Asian deletion (− -^SEA^) was seen with IVS-II-654 (C > T) in the β-globin gene in the proband’s mother(II:3). The proband was heterozygous for Hb H (−α^3.7^/−-^SEA^) and β^CD41–42^ /β^IVS-II-654^. **b**.Gap-PCR revealed a Southeast Asian deletion (− -^SEA^) for mother, the rightward deletion (−α^3.7^) for father, and Hb H disease (−-^SEA^/−α^3.7^) for the son (the proband). **c**. RDB for the 3 genotypes of the α2 gene point mutations (Hb CS, Hb QS and Hb WS) showed no point mutation. **d**. RDB assay for the 17 genotypes of the β-thalassemia point mutations

We presented a 1-year old child (Fig. 1a - III: 1) born to the non-consanguineous parents. The patient and the parents’ phenotypes were presented in Table 1 and Fig. 2a.

**Table 1:** Hematological features, Hb electrophoresis and the genotype analysis of the proband’s family

parameters	I:1	I:2	II:1	II:2	II:3	III:1	Reference
Sex/Age (years)	M/55	F/52	F/34	M/32	F/30	M/1	–
RBC (× 10^12^/L)	5.8	5.2	4.8	6.2	5.6	3.63	4.0–5.5
Hb (g/L)	141	122	125	133	118	57	120–160
MCV (fL)	82.6	65.1	80.5	63.6	64.8	48.8	82–95
MCH (pg)	28.5	21.1	30.1	21	21	15.7	27–31
MCHC (g/L)	340	338	335	333	330	322	320-3 s60
HbA2 (%)	2.9	5.2	3	5.7	5.2	3.2	2.5–3.5
HbA0 (%)	89	85.3	90.1	81.2	84.4	61.7	≥80
HbF (%)	0.8	1.2	1	1.5	0.4	35.1	0.0–2.3
RDW (%)	14	16	13	17	24	30	12–15
α genotype	-α^3.7^/αα	αα/αα	αα/αα	-α^3.7^/αα	--^SEA^ /αα	--^SEA^/−α^3.7^	–
β genotype	β^N^/β^N^	β^N^/β^CD41–42^	β^N^/β^N^	β^N^/β^CD41–42^	β^N^/β^IVS-II-654^	β^CD41–42^ /β^IVS-II-654^	–

*RBC* red blood cell, *Hb* hemoglobin, *MCV* mean cell volume, *MCH* mean cell hemoglobin, *MCHC* mean cell hemoglobin concentration, *M* male, *F* female, *β*^*N*^ normal β-globin gene, *I:1* grandfather, *I:2* grandmother, *II:1* father’s sister, *II:2* father, *II:3* mother, *III:1* proband

The proband was a 1-year-old boy who was the offspring of unrelated parents that originated from Hengyang city, Hunan Province of southern China. The pregnancy was uneventful, and he was delivered vaginally with a birth weight of 3250 g. There was no history of jaundice at birth and no obvious retardation of growth and development. He had a height of 74 cm and a weight of 9 kg. The abdomen was a little hard with mild hepatosplenomegaly. He looked pale when he was 6 months old. He had never been transfused during his growth. He was referred to the Pediatric clinic (The First Affiliated Hospital of Sun Yat-sen University, Guangzhou, China) to confirm the diagnosis after getting a cold. His body temperature was 38.6 °C. He got a cough. Routine peripheral blood counts and Hb electrophoresis were determined according to standard laboratory procedures. Clinical tests showed that he had mild hepatosplenomegaly as well as typical hematological features of hypochromic microcytosis with Hb level of 57 g/dL, a mean cell volume (MCV) of 48.8 fL, and mean cell hemoglobin (MCH) of 15.7 pg (Additional file 1). Iron deficiency was excluded. Though with normal hemoglobin concentration, the proband’s parents (Fig. 2a , II:2, II:3) showed microcythemia and hypochromia. Both of them were mild thalassemia. They were not blood transfusion dependent. His and his family members’ blood samples were collected using EDTA as anticoagulant after informed consent was obtained.

**Fig. 3:**
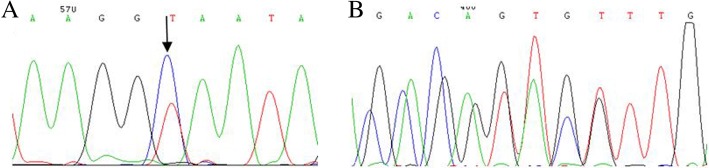
DNA sequences of proband’s β-globin genes. **a**. GCA → GTA substitution at the β-globin gene (HBB: c.316-197C > T) was observed (indicated by an arrow). **b**. Part of the reverse DNA sequencing of the β-globin gene (HBB: c.126-129delCTTT)

The hematological data was summarized in Table 1. The red cells of the proband presented severe microcytosis and hypochromia. The hemoglobin electrophoresis results showed that the percentages of HbA2 was 3.2% (house reference interval 2.5–3.5%) and Hb F was 35.1% (house reference interval 0–2.3%) (Fig. 1). Therefore, the degree of anemia, the red cell abnormalities and the clinical phenotype were inconsistent with the hemoglobin electrophoresis results. Further gap-PCR revealed a deletional Hb H (−-^SEA^/−α^3.7^) (Fig. 2b) of the proband, while the RDB assay for the α2 gene mutations indicated no mutation (Fig. 2c). The β-thalassemia point mutation RDB assay for the 17 genotypes [8] showed CD41–42(β^0^) and IVS-II-654 (β^+^) (β^CD41–42^/β^654^) (Fig. 2d). Thus, this child had Hb H (−-^SEA^/−α^3.7^) (1800 bp and 1300 bp) and β^0^/β^+^ (β^CD41–42^/β^IVS-II-654^) (Fig. 2b, Additional file 1). Then, codons 41–42(-TTCT) (HBB: c.126_129delCTTT) deletion and IVS-II-654 (C > T) (HBB: c.316-197C > T) substitution at the β-globin gene mutation were observed by Sanger sequencing (Fig. 3).

## Discussion and conclusions

In our report, the patient showed a moderate to severe anemia according to his clinical symptoms and the results of laboratory tests. Beta-thalassemia was indicated by his hematological feature. Meanwhile, the compound mutations were identified by the classic genotyping methods of β-thalassemia. The results from reverse dot blotting (RDB) for β -thalassemia point mutations suggested that it was a β^0^/β^+^ double heterozygous thalassemia, which usually presented as severe anemia and transfusion dependent [9]. However, the proband had a normal Hb A2 level (3.2%) and high Hb F level (35.1%) but no Hb H peaks on CE electrophoregram and severe microcythemia and hypochromia, which are consistent with a previous study [10]. The genotype of this patient was not consistent with phenotype, as β-thalassemia usually has a high HbA2 level [11]. The discrepancy between the genotype and phenotype made us to explore the potential reasons. A previous report about a case of the co-existence of Hb H disease (−-^SEA^/−α^4.2^) and β-thalassemia major (β^CD17A > T^/β^IVS2-654C > T^) [7], gave us a clue that our case may also have Hb H disease, which could ameliorate the phenotype of β-thalassemia via decreasing the amount of HbA2 (α_2_δ_2_). The level of HbA2 globin (3.2%) was supportive of our hypothesis. Then, Hb H disease (−α^3.7^/−-^SEA^) was verified by gap-PCR which usually screens three common α-globin deletions in south China. As a result, our patient was finally diagnosed with a compound heterozygous thalassemia [Hb H (−-^SEA^/−α^3.7^) and β^0^/β^+^ (β^CD41–42^/β^IVS-II-654^)], which is very rare in the Chinese population.

Although the patient had deletional Hb H (−-^SEA^/−α^3.7^) disease, neither Hb Bart’s nor Hb H peaks were found on the CE electropherogram. The absence of Hb H might be due to the reduction of the β-globin chain from the affected β-globin allele, leading to a less excessive β-globin chain and hence the homotetramer of the β-globin chain is minimal [9], and the absence of Hb Bart’s might be owing to increased level of Hb F. Co-inheritance of α- thalassemia could modify the phenotypes of homozygous or compound heterozygous states for β-thalassemia. The patient had low Hb levels (57 g/L), and microcythemia, hypochromia [MCV (48.8 fL) and MCH (15.7 pg)].This was consistent with a previous study, which showed that the heterozygous β- thalassemia patients who co-inherited Hb H disease, had an obvious reduction in these three hematological parameters [12]. Moreover, it had been reported that the homozygous β^0^-thal [13] and β^0^/β^+^-thal patients [14] who also had Hb H disease, had similar severe clinical manifestations when compared with those who did not have Hb H disease. Although, the ratio of α-globin and non-α-globin chain biosynthesis were completely balanced, a striking hypochromia (MCH15.0 pg) and a marked reduction of MCV (55.0 fL) were found in these patients [14, 15]. Hb H disease usually results in severe anemia and the formation of inclusion bodies and the malfunction of globins. In our report, the phenotype of Hb H disease was ameliorated by the decreasing of Hb H, as a consequence of the heterozygous β-thalassemia (β^0^/β^+^), which could decrease the Hb H inclusion bodies and hemolytic by balancing the ratio of α-globin and of β-globin. In other words, though the amount of the globins was reduced, its function was normal. We would like to speculate on the phenomena regarding the “teeter- totter” paradigm: the α-globin gene and of β-globin gene can be regarded as both ends of the “teeter-totter”. Malfunction of either of these genes would result in severe changes in the phenotype, while the malfunction of the rest of genes could rebalance the “teeter-totter”. However, re-balancing the “teeter-totter” may obscure the diagnosis of the underlying Hb H disease by reducing the phenotype on the anemia, which may increase the genetic load.

**Fig. 4:**
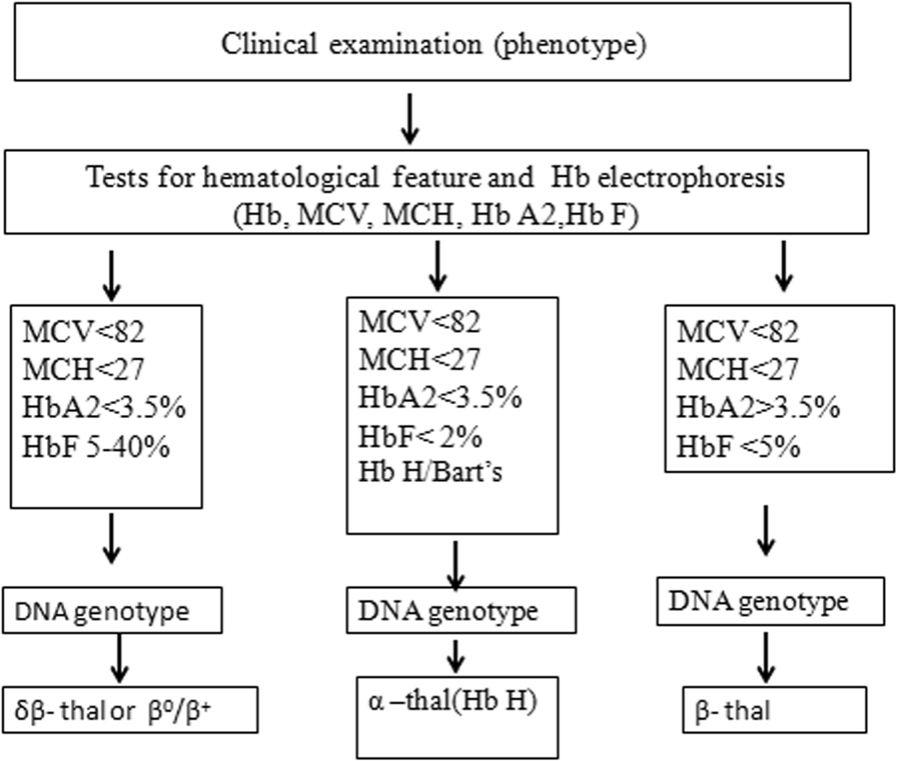
Framework for the diagnosis of a compound heterozygous thalassemia (Hb H disease and β^0^/β^+^)

Co-inheritance of a β-thalassemia defect with a single functional α-globin gene is rare. There were reports that co-inheritance of β-thalassemia trait in the Hb H disease could reduce the level of excess β-globin chains, resulting in less severe imbalance of α/β-globin chain synthesis. But if patients with β-thalassemia co-inherited HbH disease had increased hemolysis secondary to infections or fever, blood transfusion therapy may be necessary [16]. Our report was just consistent with this reference. Co-inheritance of α-thalassemia can lead to a reduction in the level of Hb A2; this does not interfere with the diagnosis of β-thalassemia carriers as the Hb A2 level in these double heterozygotes was still higher than the normal level. However, concurrent heterozygous β-thalassemia and Hb H disease could show a normal level of Hb A2 [17], and our case was consistent with this report. The diagnosis of β- thalassemia cannot be totally excluded in patients who have a normal HbA2 level, as the HbA2 level can be normal in a patient with co-inheritance of both α and β thalassemia [18]. Hb analysis alone may not be a reliable diagnostic tool, especially in patients who had concurrent Hb H disease and heterozygous β-thalassemia. Genetic analysis therefore serves as an important mean to identify patients with Hb H disease, especially in those who have unexplained phenotypes [17]. A better understanding of the interactions of β and alpha globin chains and the resulting phenotypes will help the diagnosis, prevention and controlling program of β-TM. It is also essential to design an appropriate screening strategy to detect complex mutation carriers, including those who co-inherited α- and β-globin gene defects. Together with previous reports [12–14] and our case, a valuable strategy for the diagnosis of a compound heterozygous thalassemia (Hb H disease and β^0^/β^+^) was proposed (Fig. 4).

In conclusion, we report a case who inherited the compound heterozygosity of (β^+^) IVS-II-654 and (β^0^) codons 41-42 mutations and the Hb H (--^SEA^/-α^3.7^) disease. Our examinations showed that the co-inheritance of Hb H disease could affect not only the hematological parameters (Hb, MCV and MCH) but also the HbA2 levels.

## Additional file

Additional file 1: Methods of hematological analysis and DNA analysis (DOCX 14 kb)

## Abbreviations

Hb: hemoglobin
HbF: Hemoglobin F
MCH: mean cell hemoglobin
MCHC: mean cell hemoglobin concentration
MCV: mean cell volume
PCR: Polymerase chain reaction
RBC: red blood cell
RDW: red cell distribution width
