# Supplementary material for: The phenomena of balanced effect between α-globin gene and of β-globin gene
Source: BMC Med Genet. 2018 Aug 17;19:145. doi: 10.1186/s12881-018-0659-9 (PMC6098578; doi:10.1186/s12881-018-0659-9)
Supplement: Supplementary file 1 — Methods of hematological analysis and DNA analysis (DOCX 14 kb) [file 12881_2018_659_MOESM1_ESM.docx]

**Methods**

**Hematological analysis**

Hematological data was obtained from the Sysmex XE-4000i automated blood cell counter (Sysmex, Kobe, Japan). Hb analysis was performed using an automated capillary electrophoresis system (the Capillarys 2, Sebia, France).

**DNA analysis**

Genomic DNA was extracted from the peripheral blood of the patient using a QuickGene DNA Whole Blood kit (Fujifilm, Tokyo, Japan) in accordance with the manufacturer’s instructions. We performed gap-polymerase chain reaction (gap-PCR) using a commercial genetic kit (Ya-neng Biological Products, Shenzhen LTD, China) to screen the three most common deletional thalassemias: the Southeastern Asian deletion (- -^SEA^), the rightward deletion (-α^3.7^) and the leftward deletion (-α^4.2^). We also applied a reverse dot-blot (RDB) assay (Ya-neng Biological Products, Shenzhen LTD, China) to detect the three common nondeletional α2 gene mutations: Hb Constant Spring (Hb CS, α142, Term→Gln; HBA2: c.427T>C), Hb Quong Sze [Hb QS, α125 (H8) Leu→Pro; HBA2: c.377T>C] and Hb Westmead [α122(H5)His→Gln; HBA2: c.369C>G] in a DNA Thermal Cycler ABI 9700 (ABI Company, USA). The primers used to detect the α-thalassemia genotypes and the PCR product lengths were previously described [[7](#_ENREF_7)]. Additional rare nondeletional defects were identified through Sanger sequencing of the α2- and α1-globin gene with PCR products. The 17 common nondeletional β- globin gene mutations, including CD41-42, CD71-72 and IVS-II-654, were amplified with a β-thalassemia genotype detection kit (Ya-neng Biological Products, Shenzhen LTD, China). The PCR amplifications were performed in a final volume of 25 μL containing 100 ng of genomic DNA, 100 μM of each dNTP, 0.12 μM of each primer, 0.5 U of Taq DNA polymerase (Ya-neng Biological Products, Shenzhen LTD, China),2Mm MgCl2, and 2.5 μL of 10× buffer as provided by the manufacturer. PCR was carried out with a denaturing step at 95 °C for 5 min, followed by 30 s at 94 °C, 30 s at 60 °C for 10 cycles and 30 s at 94 °C, 30 s at 56 °C, 1min at 72 °C for another 25 cycles, followed by a final 5 min at 72 °C in the thermo cycler 9700 (Applied Bio systems, Foster City, CA, USA). The quantity of PCR amplification was verified by electrophoresis on 2% agarose gel after staining with gold view. After PCR amplification, the PCR products were incubated at 42°C for 2 hours in a Hybrid Box YN-H16 (Ya-neng Biological Products, Shenzhen LTD, China) using RDB. The β-gene mutations detected were confirmed by Sanger sequencing.
